# Two cases of nodular smooth muscle proliferation suspected of primary lung cancer from preoperative images: a case report

**DOI:** 10.1186/s13019-020-01228-6

**Published:** 2020-07-22

**Authors:** Toshihiro Ikeda, Tetsuhiko Go, Kyuichi Kadota, Emi Ibuki, Hiroyasu Yokomise

**Affiliations:** 1 Faculty of Medicine, Department of General Thoracic Surgery, Breast and Endocrinological Surgery, Kagawa University, 1750-1 Ikenobe, Kita-gun, Miki-cho, Kagawa 761-0793 Japan; 2 Faculty of Medicine, Department of Diagnostic Pathology, Kagawa University, 1750-1, Ikenobe, Miki-cho, Kagawa 761-0793 Japan

**Keywords:** Nodular smooth muscle proliferation, Leiomyoma, Lung benign tumor, Case report

## Abstract

**Background:**

It is difficult to obtain a definitive diagnosis for nodular smooth muscle proliferation (NSMP) before surgery, and a pathological diagnosis is necessary to differentiate it from primary lung cancer. We report two cases of NSMP that were suspected to be primary lung cancer on preoperative images.

**Case presentation:**

Case 1: An 81-year-old man who had undergone right upper lobectomy for lung cancer 2 years earlier was point out a nodular shadow with ground glass opacity (GGO) in the lower right lobe, suggesting a second primary lung cancer by chest computed tomography (CT). A thoracoscopic partial resection of the right lower lobe was performed, and pathological diagnosis was NSMP. The patient was discharged without any problems at 3 days postoperatively.

Case 2: A 72-year-old woman was pointed out a nodular shadow suspected primary lung cancer in the left lower lobe by chest CT. Therefore, thoracoscopic partial resection of the left lower lobe was performed, and pathological diagnosis was NSMP. The patient was discharged without any problems at 5 days postoperatively.

**Conclusion:**

This report demonstrates that NSMP can be distinguished from leiomyoma and hamartoma by imaging features and pathological findings.

## Background

Benign Lung tumors are rare, ranging from 2 to 5% of all lung tumors. Smooth muscle lesions originating in the lung represent 2% of benign lung tumors [1]. Nodular smooth muscle proliferation (NSMP) has been described in detail elsewhere [2]. However, no published reports included in PubMed have examine the radiological and pathological findings of NSMP. Here, we report two cases of NSMP that required differentiation from primary lung cancer.

## Case presentation

### Case report:1

**Fig. 1:**
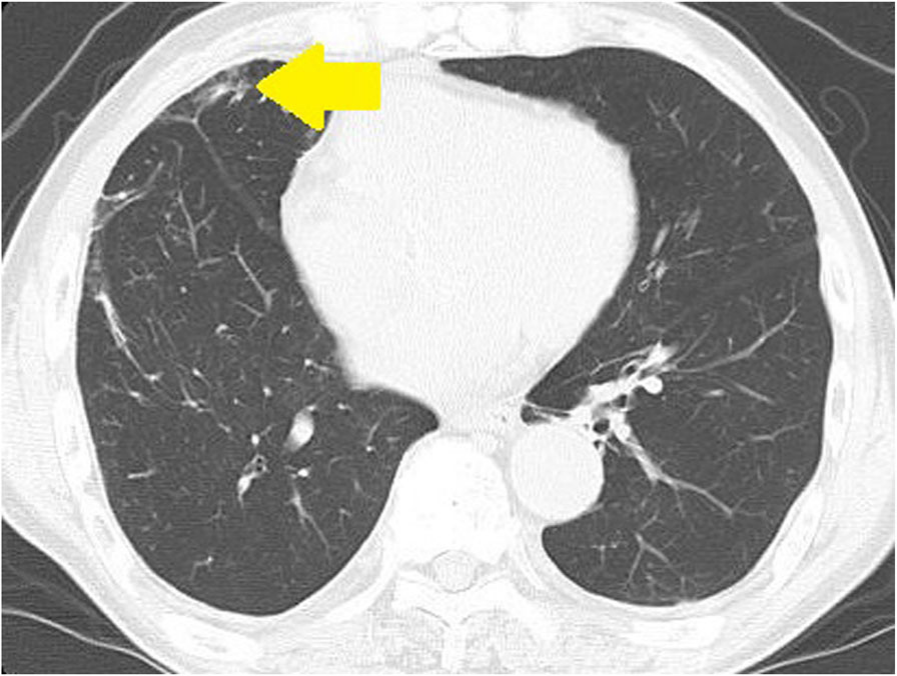
Chest computed tomography. a 12-mm right lower lobe nodule with ground glass opacity

**Fig. 2:**
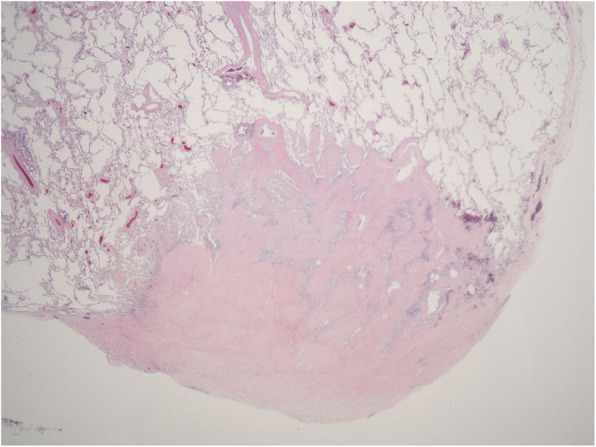
Pathological findings. Dendritic growth of smooth muscle tissue seen, with an unclear boundary between normal tissue and the lesion (Hematoxylin and Eosin stain × 20)

An 81-year-old man had undergone a right upper lobectomy for right upper lobe lung cancer. Two years postoperatively, chest computed tomography (CT) revealed a nodular shadow with ground glass opacity (GGO) in the lower right lobe, suggesting a second primary lung cancer, although the patient was asymptomatic (Fig. 1). ^18^F-fluorodeoxyglucose - positron emission tomography (^18^FDG-PET) was not performed preoperatively because the lesion was small. The patient consented to a thoracoscopic partial resection of the right lower lobe for diagnosis and treatment. Pathological findings revealed a white-colored, 10-mm nodule with an unclear boundary just below the pleura. Hematoxylin and eosin staining revealed spindle-shaped cells with eosinophilic cytoplasm that had proliferated in a dendritic manner (Fig. 2). Immunohistochemistry revealed desmin and alpha-smooth muscle actin positivity. A diagnosis of NSMP was made. No postoperative recurrence has been observed without additional postoperative treatment.

### Case report:2

**Fig. 3:**
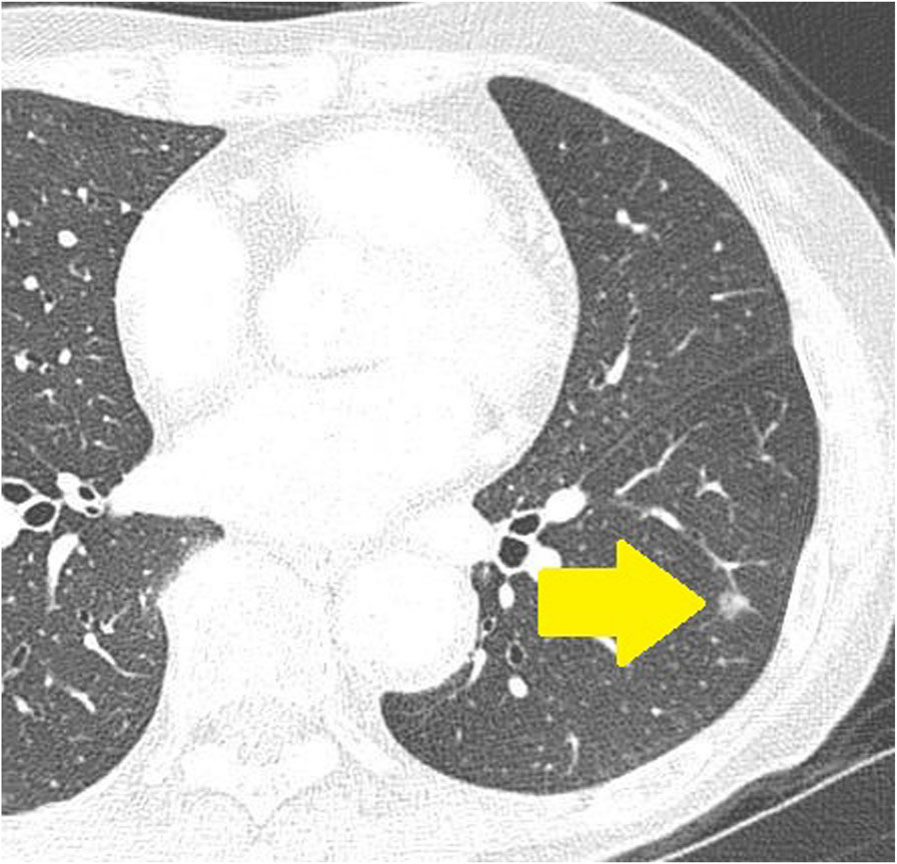
Chest computed tomography. a 6-mm left lower lobe nodule with ground glass opacity

**Fig. 4:**
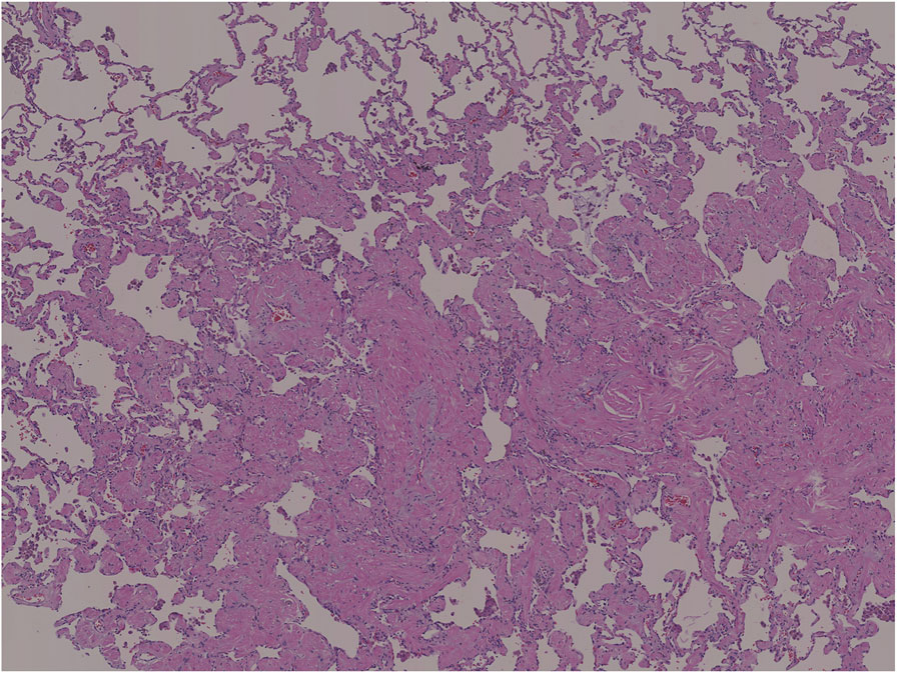
Pathological findings. Dendritic growth of smooth muscle tissue (Hematoxylin and eosin stain, × 20)

A 72-year-old woman with no symptoms had a medical checkup, and a subsequent CT revealed a nodular shadow in the left lower lobe (Fig. 3). ^18^FDG-PET was not performed preoperatively because the lesion was small. We suspected a primary lung cancer, and the patient consented to a thoracoscopic partial resection of the left lower lobe for diagnosis and treatment. Pathological findings revealed a 5-mm nodule with an unclear boundary. Hematoxylin and eosin staining revealed collagen fibers, bronchiolar metaplasia, lymphocyte-induced inflammatory cell infiltration, and smooth muscle dendritic growth with foamy histocytes (Fig. 4), Immunohistochemistry revealed desmin and alpha-smooth muscle actin positivity. A diagnosis of NSMP was made. The patient received no postoperative treatment, and no recurrence has been observed.

## Discussion/conclusion

The histopathological features of NSMP resemble those of hamartoma and leiomyoma [2], as all three lesions exhibit a proliferation of spindle-shaped cells and smooth muscle upon hematoxylin and eosin staining. Although an immunohistochemical analysis of NSMP reveals desmin and SMA positivity, this is not useful for differentiation from hamartoma or leiomyoma. The difference is that NSMP lesions contain more smooth muscle compared to hamartomas. NSMP also differs from leiomyoma in lesion size. Although no specific definition or standard of NSMP has been established, pathological findings indicate that NSMP exhibits dendritic smooth muscle proliferation, which results in unclear edges. This margin type clearly differs from the smooth margins of leiomyomas and hamartomas. In our cases, the smooth muscle tissue within the lesions appeared dendritic, with an undefined border. In addition, no tissue growth other than smooth muscle tissue was observed in the lesions, which was unlike a hamartoma. These pathological findings led us to diagnose NSMP in both cases. We speculated that the GGO observed around the nodules on preoperative CT was due to the spread of the smooth muscle tissue in a dendritic manner.

A previous report identified fatal pulmonary hypertension as a complication associated with diffuse smooth muscle proliferation on the lungs, which results from pulmonary artery distortion and occlusion by smooth muscle tissue and localized thrombosis [3]. In both our cases, pulmonary hypertension or symptoms of the right heart failure, such as dyspnea, palpitation and edema, were not observed because smooth muscle proliferation was localized, rather than diffuse. However, additional research is needed to determine whether localized smooth muscle proliferation affects pulmonary hypertension. Both patients in this report underwent a partial resection, and neither received postoperative treatment or developed a recurrence. However, some reports of smooth muscle lesions have described malignant transformation [4] and high malignancy [5]. Furthermore, the postoperative follow-up period remains controversial. Further studies are required to understand the treatment and postoperative strategies in managing NSMP. In conclusion, this report demonstrates that NSMP can be distinguished from leiomyoma and hamartoma by imaging features and pathological findings. To our knowledge, this is the first report to review the radiological and pathological findings of NSMP, and it will serve to improve patient outcomes.

## Data Availability

Not applicable.

## Abbreviations

CT: computed tomography
GGO: ground glass opacity
^18^FDG-PET: ^18^F-fluorodeoxyglucose - positron emission tomography
NSMP: nodular smooth muscle proliferation

## References

[1] White SH, Ibrahim NB, Forrester-Wood CP, Jeyasingham K (1985). Leiomyomas of the lower respiratory tract. Thorax..

[2] Fraire AE, Dail DH, Tomashefski JF, Cagle PT, Farver CF, Fraire AE (2008). Mesenchymal tumor, part I. Dail and Hammar’s pulmonary pathology, volume II: neoplastic lung disease.

[3] Kay JM, Kahana LM, Rihal C (1996). Diffuse smooth muscle proliferation of the lungs with severe pulmonary hypertension. Hum Pathol.

[4] Ogawa M, Hara M, Ozawa Y, Moriyama S, Yano M, Shimizu S (2011). Benign metastasizing leiomyoma of the lung with malignant transformation mimicking mediastinal tumor. Clin Imaging.

[5] Qin BD, Jiao XD, Zang YS (2018). Primary pulmonary leiomyosarcoma: a population-based study. Lung Cancer.

